# Ultra-wide field imaging to assess the optic nerve and retina in Boston type I and II keratoprosthesis patients

**DOI:** 10.1186/s40662-022-00289-z

**Published:** 2022-05-07

**Authors:** William R. Bloom, Matthew D. Karl, Sarah B. Smith, Yusra F. Shao, William Terrell, Ahmad B. Tarabishy, Andrew J. Hendershot, Rebecca A. Kuennen, Tyler D. Oostra, Thomas F. Mauger, Colleen M. Cebulla

**Affiliations:** Department of Ophthalmology and Visual Sciences, Havener Eye Institute, The Ohio State University Wexner Medical Center, 915 Olentangy River Road Suite 5000, Columbus, OH 43212 USA

**Keywords:** Corneal diseases, Diagnostic eye imaging, Posterior eye segment, Prosthesis implantation

## Abstract

**Background:**

The ability to view the posterior segment in keratoprosthesis (Kpro) implanted patients is limited. The purpose of this retrospective, observational study was to investigate the use of ultra-wide field (UWF) scanning laser ophthalmoscopy imaging and its utility for serial evaluation of the retina and optic nerve in patients with either a Boston type I or II Kpro.

**Methods:**

A retrospective chart review was performed for patients with a Boston type I or II Kpro seen at The Ohio State University Wexner Medical Center. Images were graded for quality by two masked observers on a defined four-point scale (“Poor”, “Fair”, “Good”, or “Very good”) and assessed for visible posterior segment anatomy. Interobserver agreement was described using the Kappa statistic coefficient (κ) with 95% confidence intervals.

**Results:**

A total of 19 eyes from 17 patients were included in this study. Eighteen eyes had a type I Kpro, while one eye had a type II Kpro. UWF imaging from 41 patient visits were reviewed by two observers. Interobserver agreement between the two graders was fair for image quality (κ = 0.36), moderate for visibility of the macula with discernible details (κ = 0.59), moderate for visibility of the anterior retina with discernable details (κ = 0.60), and perfect agreement for visibility of the optic nerve with discernible details (κ = 1.0). In 6 eyes, UWF imaging was performed longitudinally (range 3–9 individual visits), allowing for long-term follow-up (range 3–46 months) of posterior segment clinical pathology.

**Conclusions:**

UWF imaging provides adequate and reliable visualization of the posterior segment in Kpro implanted patients. This imaging modality allowed for noninvasive longitudinal monitoring of retinal and optic nerve disease in this selected patient population.

## Background

Artificial corneal transplantation was developed to treat severe ocular surface disease and corneal blindness in cases of poor prognosis with traditional penetrating keratoplasty. The Boston type I keratoprosthesis (Kpro) is the most used artificial corneal design worldwide [1]. The less commonly used Boston type II Kpro is indicated in patients with severe, end-stage corneal disease and implanted through surgically closed eyelids [2, 3].

The ability to view the posterior segment in Kpro implanted patients is limited due to poor corneal health and ocular comorbidities. Standard fundus examination and photography methods are often unrevealing and provide little clinical utility after Kpro implantation. Other factors that complicate adequate visualization and monitoring of the posterior segment in these patients include the small, three millimeter optic aperture, the presence of a bandage contact lens (BCL), and the development of a retroprosthetic membrane (RPM) [4]. Furthermore, common indications for Kpro implantation, such as aniridia and limbal stem cell deficiency, often result in nystagmus and other fixation difficulties [5]. The inability to fixate makes standard imaging particularly difficult.

Despite these difficulties, evaluating for posterior segment clinical pathology in Kpro implanted eyes is essential since optic nerve and retinal disease are quite prevalent in this patient population. Many patients receiving Kpro implantation have posterior segment disease that requires long-term follow-up [6–9]. In addition, post-surgical complications may necessitate longitudinal monitoring to avoid further visual morbidity [9–11]. The purpose of this retrospective, observational study was to investigate the use of ultra-wide field (UWF) scanning laser ophthalmoscopy imaging and its utility for serial evaluation of the retina and optic nerve in patients with either a Boston type I or II Kpro.

## Methods

This was a single-institution, retrospective, observational chart review investigating the clinical utility of UWF imaging in Kpro implanted patients. Patient demographics, ocular comorbidities, Kpro implant type, visual acuity, and UWF imaging characteristics were recorded and analyzed as part of this investigation. This study was performed in accordance with the principles outlined by the Declaration of Helsinki and conformed to the Health Insurance Portability and Accountability Act of 1996 regulations. The Ohio State University Institutional Review Board approved this study.

Patients were identified through medical record chart review. Those patients seen at The Ohio State University Wexner Medical Center with either a Boston type I or II Kpro between January 1, 2009, and October 1, 2020, were consecutively enrolled and retrospectively reviewed as part of this study. Patients were included if they had a Boston type I or II Kpro implantation for any indication and subsequently underwent UWF imaging as part of their clinical care. Those Kpro patients without UWF imaging in their clinical record were excluded from review. Patients with multiple visits including UWF imaging sessions were analyzed longitudinally.

As part of this research study, images obtained during clinical visits using the Optos scanning laser ophthalmoscope imaging system (California or 200Tx; Optos, Marlborough, MA) were reviewed independently by two retina fellows. Both observers were masked to the clinical data and history of the patients. Each observer independently graded the UWF images using a standard scale. Image quality was graded on a four-point scale as “Poor”, “Fair”, “Good”, or “Very good”. Observers were instructed to use the following guidelines for the purposes of image quality grading—Poor: incomplete optic disc or blurred posterior segment structures without meaningful clarity; Fair: image provided moderate structural resolution but did not reveal the entire posterior pole; Good: clear image with view of the entire posterior pole; Very good: excellent resolution with field of view to equatorial retina. In addition, observers noted if the optic nerve, macula, and/or anterior retina were visible in each respective image with discernible details. For the purpose of this report, the anterior retina was defined as visualization of one or more quadrants outside the vortex vessels. Observers were permitted to use the software-integrated zoom tool to assist in image evaluation. Images were typically viewed at 100% with occasional magnification up to 200%, as indicated. The outcome measures of interest included the reliability of UWF imaging to view the posterior segment and the clinical utility of UWF imaging as a method to follow clinical pathology in Kpro patients. Image quality and visible posterior segment anatomy were used to assess clinical utility of UWF imaging in Kpro patients.

### Statistical analysis

Results were expressed as mean ± standard deviation (SD) for continuous variables and as raw counts with associated proportions for categorical variables. Clinical characteristics were analyzed using descriptive statistics. Crude agreement between observers was calculated and represented the raw counts and associated proportions where observers demonstrated exact agreement on a particular parameter. Interobserver agreement was also assessed using the Kappa statistic coefficient (κ) ± standard error with 95% confidence intervals (CI). Levels of agreement were evaluated using the interpretation suggested by Landis and Koch: < 0.20, slight; 0.21–0.40, fair; 0.41–0.60, moderate; 0.61–0.80, substantial; and 0.81–1.00, almost perfect agreement [12]. Unlike crude agreement, which does not account for potential observer guessing, the Kappa statistic takes into consideration agreement due to random chance [13]. Analyses were performed using JMP Pro, v. 15.2.0 (SAS Institute Inc., Cary, NC, 1989–2021).

## Results

### Patient characteristics

**Fig. 1:**
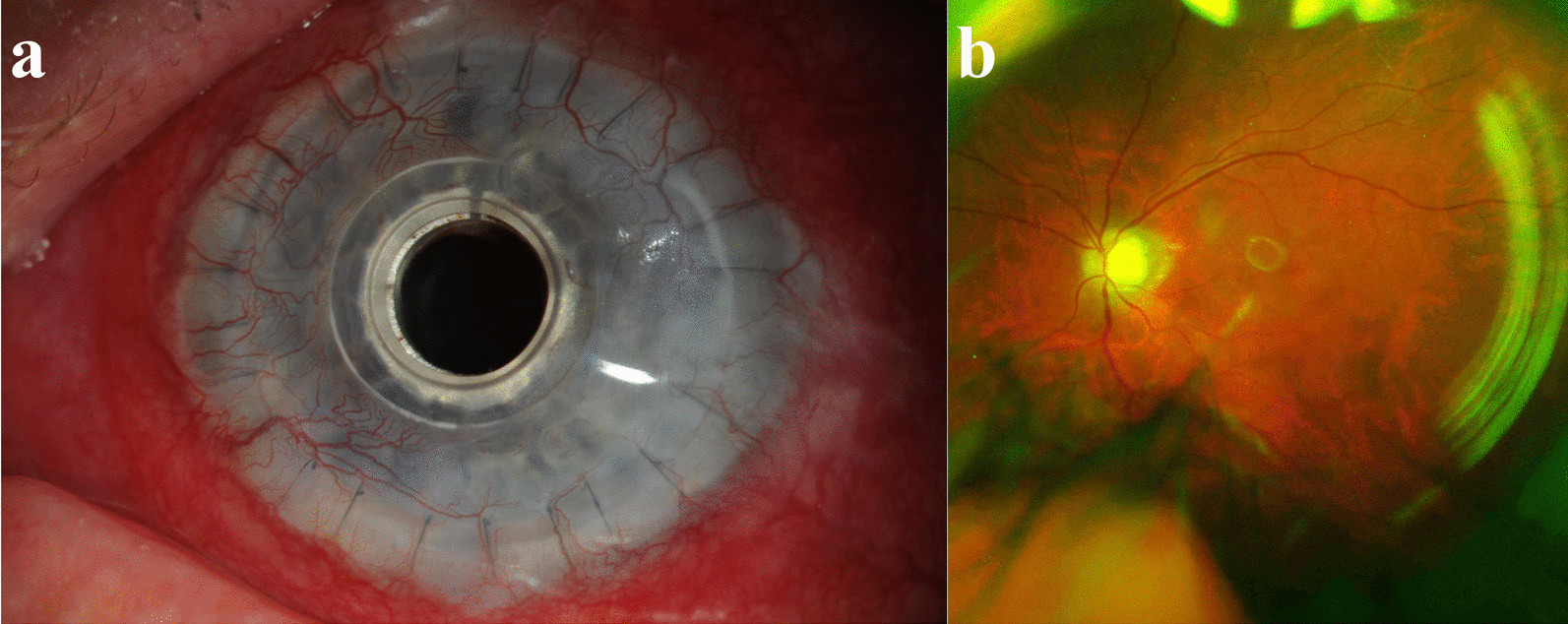
Patient with a history of severe rosacea keratoconjunctivitis and a Boston type I Kpro. **a** External photography demonstrating Kpro and conjunctival hyperemia. **b** Ultra-wide field imaging of the posterior segment demonstrating slight optic nerve pallor and an attached retina

**Fig. 2:**
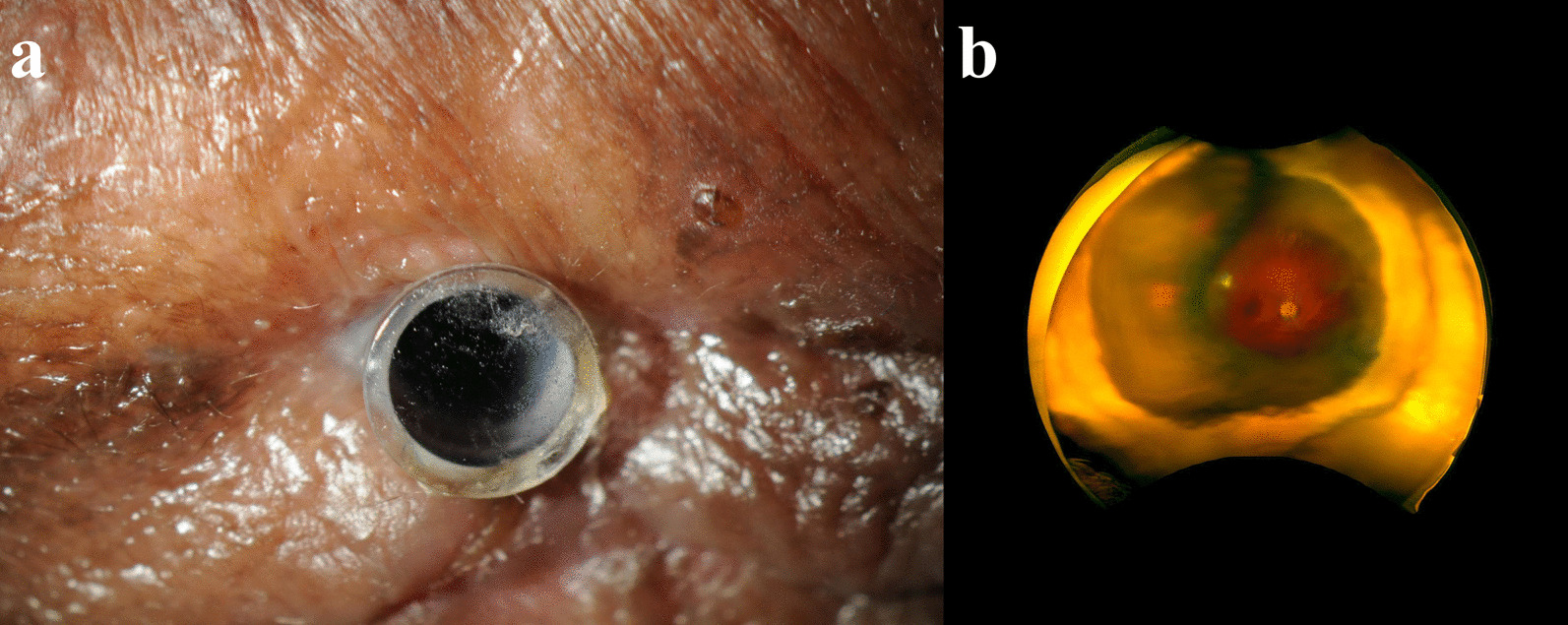
Patient with a history of ocular cicatricial pemphigoid, glaucoma, and a Boston type II Kpro. **a** External photography demonstrating type II Kpro. **b** Ultra-wide field imaging of the posterior segment demonstrating an optic nerve without signs of edema or pallor and no evidence of retinal disease in the available view

Nineteen eyes from 17 patients implanted with a Boston type I or II Kpro were enrolled into the study. Demographic and clinical characteristics are shown in Table 1. Images from 41 individual patient visits were analyzed. The mean age at the time of first imaging was 62.5 ± 16.5 years and the age range was from 29 to 86 years. Most of the enrolled eyes belonged to female patients (73.7%). Eighteen eyes (94.7%) were implanted with a type I Kpro. The most common preoperative diagnosis was a previous failed corneal transplant, which occurred in 63.2% of patients. One eye (5.3%) was implanted with a type II Kpro due to a preoperative diagnosis of ocular cicatricial pemphigoid. Figures 1 and 2 show representative images for types I and II Kpro patients, respectively.

**Table 1:** Demographic and clinical characteristics

Characteristic	All eyes n = 19
Age in years, mean (SD)	62.5 (16.5)
Sex, n (%)	
Female	14 (73.7)
Male	5 (26.3)
Implant type, n (%)	
Kpro I	18 (94.7)
Kpro II	1 (5.3)
Preoperative diagnoses, n (%)	
Previous failed corneal transplant	12 (63.2)
Corneal opacity	8 (42.1)
Aphakia	8 (42.1)
Corneal edema	8 (42.1)
Glaucoma	5 (36.8)
Historical retinal detachment with silicone oil	5 (26.3)
Aniridia	3 (15.8)
Congenital glaucoma	3 (15.8)
Hypotony	2 (10.5)
Vascularized corneal scar	2 (10.5)
Ocular cicatricial pemphigoid	2 (10.5)
Symblepharon	2 (10.5)
Limbal stem cell deficiency	2 (10.5)
Scleral thinning	1 (5.3)
Phthisis bulbi	1 (5.3)
Dislocated lens implant	1 (5.3)
Perforated acanthamoeba corneal ulcer	1 (5.3)
Corneal scarring	1 (5.3)
Nuclear sclerotic cataract	1 (5.3)
Rosacea related keratitis	1 (5.3)
Age-related macular degeneration	1 (5.3)
Buphthalmos	1 (5.3)
Corneal thinning	1 (5.3)

*SD=* standard deviation; *Kpro=* keratoprosthesis

Patient visit, UWF imaging, and longitudinal follow-up characteristics are shown in Table 2. The presence of potential UWF imaging obstacles was collected and analyzed. In 92.7% of images, the patient was wearing a BCL, while 12.2% of images were in patients with an RPM. A preponderance of images (80.5%) was captured in patients with visual acuity of 20/100 or worse; 56.1% of images were in eyes with counting fingers, hand motion, or light perception vision. The most common posterior segment indication for UWF imaging was glaucoma in 14 eyes (73.7%). A total of nine eyes (47.4%) had either a history or an active retinal condition. This included a history of retinal detachment in four eyes (21.1%), cystoid macular edema (CME) in two eyes (10.1%), and nonexudative age-related macular degeneration (AMD) in two eyes (10.5%). Other retinal conditions included exudative AMD, a history of recurrent uveitis, a history of fungal endophthalmitis, epiretinal membrane (ERM), and subretinal fibrosis. The records of 12 eyes (63.2%) described a limited or poor view of the posterior segment on clinical examination associated with the Kpro implant.

**Table 2:** Patient visit, ultra-wide field imaging, and longitudinal follow-up characteristics

Characteristic	
Posterior segment indications for initial UWF imaging, n (%)	All eyes n = 19
Glaucoma	14 (73.7)
History of retinal detachment	4 (21.1)
Cystoid macular edema	2 (10.5)
Nonexudative AMD	2 (10.5)
Exudative AMD	1 (5.3)
History of recurrent uveitis	1 (5.3)
History of fungal endophthalmitis	1 (5.3)
Epiretinal membrane	1 (5.3)
Subretinal fibrosis	1 (5.3)
Visual acuity, n (%)	Visits with UWF images n = 41
Better than 20/100	8 (19.5)
20/100 to 20/400	10 (24.4)
CF	9 (22.0)
HM	11 (26.8)
LP	3 (7.3)
Imaging Obstacles, n (%)	
BCL	28 (68.3)
BCL and RPM	4 (9.8)
BCL and PCO	2 (4.9)
BCL and silicone oil	2 (4.9)
None	2 (4.9)
BCL and nystagmus	1 (2.4)
BCL and precipitates on posterior KPro	1 (2.4)
RPM	1 (2.4)
Posterior segment indications for follow-up UWF imaging, n (%)	Eyes with longitudinal follow-up n = 6
Glaucoma	6 (100)
Nonexudative AMD	2 (33.3)
History of retinal detachment	1 (16.7)
History of fungal endophthalmitis	1 (16.7)
Epiretinal membrane	1 (16.7)
Subretinal fibrosis	1 (16.7)

*UWF=* ultra-wide field; *AMD=* age-related macular degeneration; *CF=* counting fingers; *HM=* hand motion; *LP=* light perceptions; *BCL=* bandage contact lens; *RPM=* retroprosthetic membrane; *PCO=* posterior capsule opacification; *Kpro=* keratoprosthesis

Six eyes from four patients were evaluated longitudinally using UWF imaging. The follow-up period in patients with longitudinal evaluation ranged from 3 to 46 months between the first and last visit where UWF imaging was performed. In patients with longitudinal follow-up, the total number of individual visits where UWF imaging was performed ranged from 3 to 9 visits. Longitudinal UWF imaging was used to monitor patients with glaucoma, nonexudative AMD, a history of retinal detachment, a history of fungal endophthalmitis, ERM, and subretinal fibrosis.

**Fig. 3:**
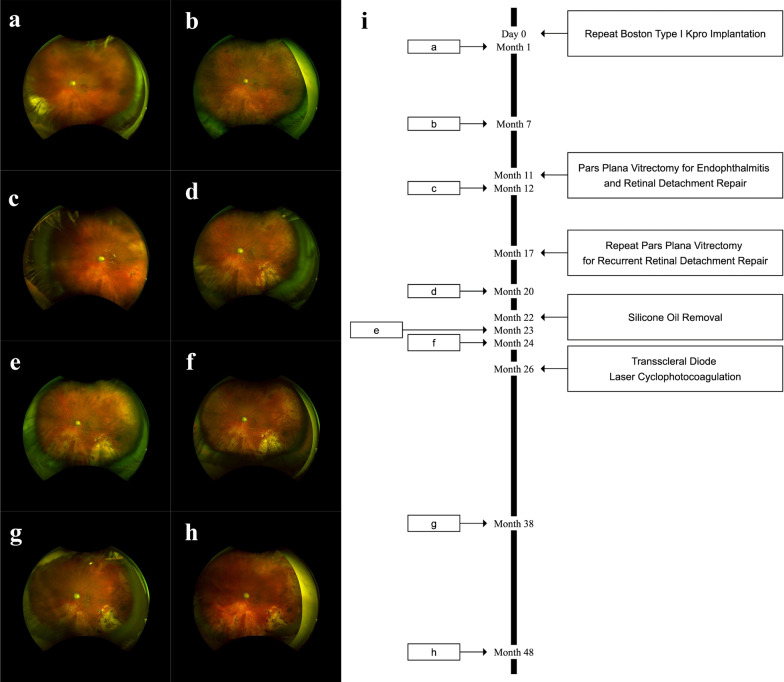
Longitudinal ultra-wide field imaging and timeline of imaging and interventions in a patient with a history of limbal stem cell deficiency after undergoing repeat Boston type I Kpro implantation. Initial ultra-wide field imaging showed a nevus temporally and follow-up imaging showed the development of a chorioretinal scar in the inferotemporal region. **a** Month 1 follow-up visit. **b** Month 7 follow-up visit. **c** Month 12 follow-up visit. **d** Month 20 follow-up visit. **e** Month 23 follow-up visit. **f** Month 24 follow-up visit. **g** Month 38 follow-up visit. **h** Month 48 follow-up visit. **i** Timeline of imaging and interventions after repeat type I Kpro implantation. Boxes **a**–**h** on the left correspond to the respective ultra-wide field image and when the image was taken during the follow-up period. Surgical interventions during the follow-up period are shown on the right

Follow-up imaging and a timeline of imaging and interventions in a patient with limbal stem cell deficiency is shown in Fig. 3. Prior to the first UWF imaging visit, the patient previously had a Boston type I Kpro, which was subsequently removed after developing fungal endophthalmitis and keratitis. Following two failed penetrating keratoplasties, a repeat type I Kpro was indicated after resolution of the infection. During the follow-up period, the patient subsequently underwent pars plana vitrectomy (PPV) for a recurrence of endophthalmitis and retinal detachment repair, a repeat PPV for a recurrent retinal detachment repair, silicone oil removal, and transscleral diode laser cyclophotocoagulation for uncontrolled secondary glaucoma.

### Image quality and visible posterior segment anatomy

**Fig. 4:**
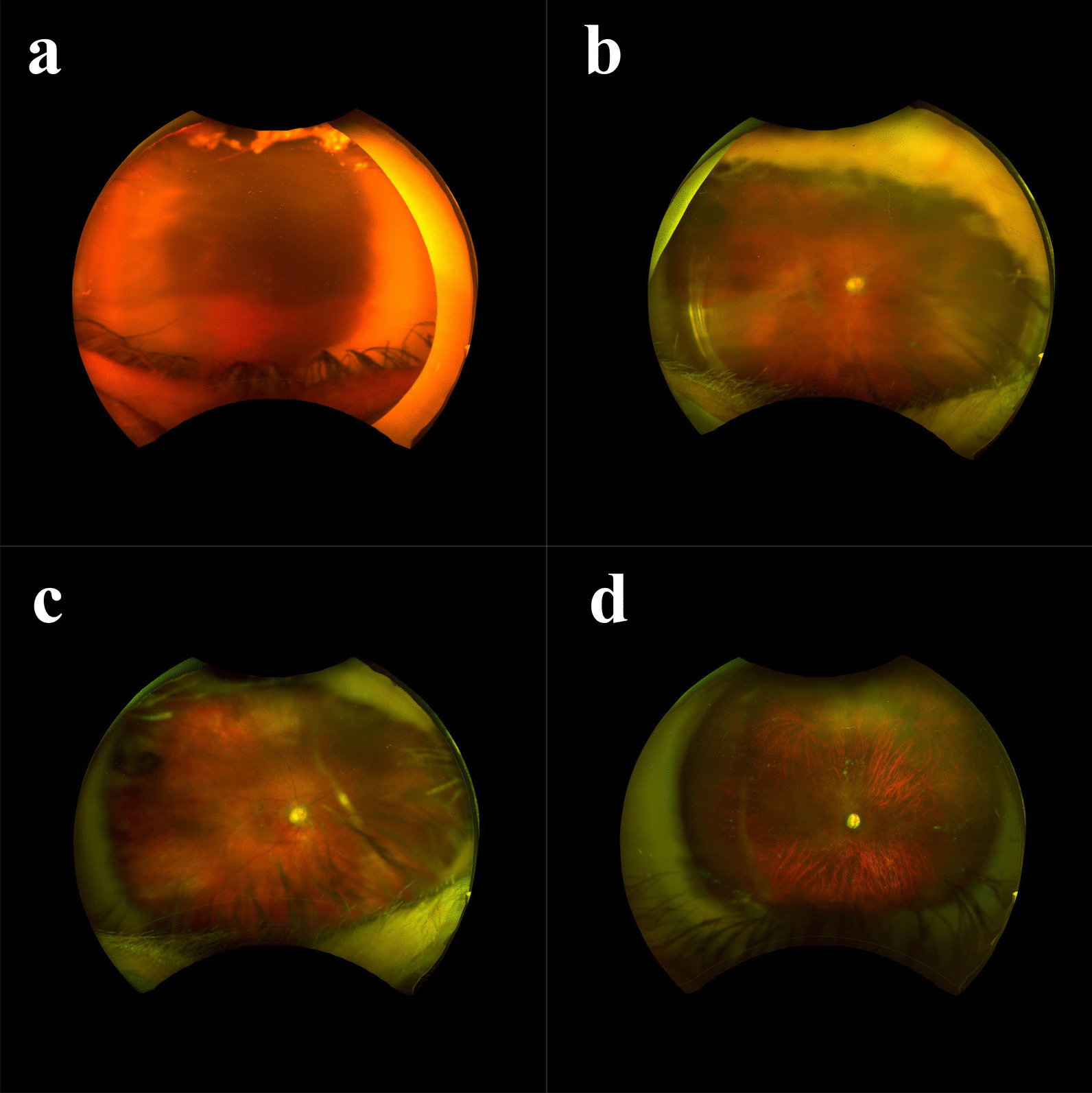
Representative images where there was full agreement on image quality using a standard scale between the two observers. **a** “Poor” image that was considered ungradable by both observers. **b** “Fair” image. **c** “Good” image. **d** “Very good” image

The imaging quality and visible posterior segment anatomy with discernable details were similar between observers (Table 3). Observer 1 graded three images (7.3%) as “Poor” quality, while observer 2 graded five images (12.2%) as “Poor” quality. Of the 41 images, observer 1 graded 38 images (92.7%) as “Fair”, “Good”, or “Very good” quality. Observer 2 graded 36 images (87.8%) as “Fair”, “Good”, or “Very good” quality. Only one “Poor” image was deemed to be completely ungradable by both observers. Of all the 41 images, there was crude agreement on image quality on 23 images (56.1%). Representative “Poor”, “Fair”, “Good”, and “Very good” images with full (100%) agreement between the two observers are shown in Fig. 4.

**Table 3:** Ultra-wide field imaging quality and visible anatomy by observer

Characteristic	Observer 1 n = 41	Observer 2 n = 41	Crude agreement n = 41
Image quality, n (%)			23 (56.1)
Poor	3 (7.3)	5 (12.2)	
Fair	18 (43.9)	18 (43.9)	
Good	10 (24.4)	10 (24.4)	
Very good	10 (24.4)	8 (19.5)	
Anatomy visible with discernible details, n (%)			
Optic nerve	40 (97.6)	40 (97.6)	41 (100)
Macula	34 (82.9)	33 (80.5)	36 (87.8)
Anterior retina	18 (43.9)	18 (43.9)	33 (80.5)

Both observers noted that the optic nerve was visible with discernible details in 40 images (97.6%) and the anterior retina visible with discernible details in 18 images (43.9%). In addition, both observers found the macula to be visible with discernable details in more than 80% of images. There was crude agreement of 100%, 87.8%, and 80.5% with respect to visualization with discernible details of the optic nerve, macula, and anterior retina, respectively.

### Kappa interobserver agreement analysis

The kappa statistic coefficients assessing interobserver agreement are shown in Table 4. There was fair agreement (κ = 0.36, 95% CI 0.14 to 0.59) for imaging quality, moderate agreement (κ = 0.59, 95% CI 0.27 to 0.91) for visibility of the macula with discernible details, and moderate agreement (κ = 0.60, 95% CI 0.36 to 0.85) for visibility of the anterior retina with discernable details between the two observers. There was perfect agreement (κ = 1.0) between the two observers for visibility of the optic nerve with discernable details.

**Table 4:** Kappa statistic coefficient for two observers

Characteristic	Kappa statistic coefficient (κ)	Standard error	95% Confidence interval
Image quality	0.36	0.11	0.14 to 0.59
Visible optic nerve with discernible details	1.0	*	*
Visible macula with discernible details	0.59	0.16	0.27 to 0.91
Visible anterior retina with discernible details	0.60	0.13	0.36 to 0.85

*Not Applicable, perfect agreement

*κ=* Kappa statistic coefficient

## Discussion

In this study, UWF imaging provided adequate visualization of the posterior segment in Kpro implanted patients. This imaging modality allowed for noninvasive longitudinal monitoring of retinal and optic nerve clinical pathology in this patient population. Serial visualization and documentation of glaucomatous optic discs and peripheral retinal diseases were successfully achieved by incorporating UWF imaging. Poor vision, ocular comorbidities, and the development of postoperative complications may impact image acquisition and quality in this sample of patients. In addition, since different technicians may have acquired images, the quality of images could also have been influenced by the technician’s skill. Images may therefore be better or worse depending on the patient, the person taking the images, and when the image was acquired. Despite these differences many clinically useful images were captured.

Visualization of posterior segment structures in Kpro patients is often difficult with indirect ophthalmoscopy and with standard fundus photography due to patient-specific ocular comorbidities, post-surgical complications, and the small aperture of the Kpro implant [6, 7, 14–16]. The results of this study support existing data suggesting UWF imaging provides meaningful clinical utility in the diagnosis and follow-up of posterior segment disease in Kpro patients. In a case series of 10 Kpro-implanted patients, Kornberg et al. found that UWF imaging detected 100% of pathology identified on clinical examination [17]. The authors also reported improved detection of posterior segment disease compared with clinical examination in patients with RPM, a complication seen in up to 65% of Kpro implanted patients [9, 15, 17]. In our study, one image from an RPM patient was rated as “Poor” and deemed ungradable by both observers. The remaining four images from patients with a RPM provided at least some degree of clinically meaningful information. Imaging obstacles, such as a RPM or the presence of silicone oil, appeared to impact the quality of some images. In addition, capturing the anterior retina in all four quadrants simultaneously was difficult to achieve in this patient population. These findings are consistent with those reported in past studies [17–19]. Employing eye steering techniques, changing patient positioning, and acquiring multiple images in a single imaging session may improve the success rate of high quality images in this patient population [20–23].

Velez-Montoya et al. investigated the interobserver agreement of UWF imaging in a sample of 13 type I Kpro patients [18]. In their prospective study, patients underwent indirect ophthalmoscopy by two experienced retina specialists who then completed a 30-question questionnaire assessing posterior segment clinical pathology and anatomy. Afterwards, a minimum of three UWF images were taken for each patient. Forty-eight hours after the initial examination, the same retina specialists repeated the questionnaire using the UWF images. The authors reported moderate to high interobserver reliability when evaluating posterior segment anatomy with UWF imaging, which was consistent with the findings of our study [18]. Furthermore, we found moderate to high agreement when evaluating posterior segment anatomy between two retina fellow observers, providing additional support of UWF imaging use in this patient population.

Past studies investigating the use of UWF imaging in Kpro implanted patients included only the type I Kpro [17, 18]. In this report we included the use of UWF imaging in a patient implanted with a type II Kpro. In this patient, some anatomic structures, such as the optic nerve, were visible with meaningful clarity with UWF imaging. This was significant as this patient had a history of glaucoma that could no longer be assessed upon clinical examination as there was no view of the posterior segment.

In difficult to image patient populations, UWF imaging was found to be a superior modality for viewing the posterior segment compared to clinical examination and standard fundus photography [24]. Previous studies have evaluated the sensitivity and specificity to detect retinal lesions using UWF imaging in various patient populations [22, 23, 25, 26]. We did not evaluate the sensitivity and specificity of UWF imaging to dilated fundus examination or standard fundus photography in this study. While dilated fundus examination is considered the gold standard for detection of posterior segment disease, in our cohort of Kpro patients, there was often no view of the posterior segment on clinical examination and standard fundus photography was not routinely performed due to low clinical utility [22]. It would be difficult to determine the sensitivity and specificity of UWF imaging to detect posterior segment pathology in the Kpro patient population due to the lack of a reliable and valid standard of care [18].

A recent report of 169 type I Kpro implanted eyes found posterior segment complications using a combination of clinical examination, B-scan ultrasonography, and optical coherence tomography in approximately 40% of eyes, with the highest incidence complications being ERM (16.6%), CME (12.4%), vitreitis (11.2%), retinal detachment (9.5%), and endophthalmitis (4.1%) [16]. Chew et al. reported preexisting glaucoma in 73% of Kpro patients with increased intraocular pressure (38%) and glaucoma progression (14%) being two common postoperative complications after Kpro implantation [8]. Neither study included the use of standard fundus photography or UWF imaging as a means to assist in the diagnosis or monitoring of posterior segment disease. Here, 73.7% of eyes had glaucoma and 47.4% of eyes had a retinal condition at the time of UWF imaging. Visualization of the optic nerve, macula, and anterior retina with discernable details was possible using UWF imaging alone with demonstrated agreement between observers. Longitudinal imaging facilitated management of complex ocular conditions, including retinal detachment and secondary glaucoma following the reimplantation of a type I Kpro in the setting of fungal endophthalmitis and failed penetrating keratoplasties.

A limitation of this study was the lack of a formal observer training period and no expert consensus scale for image quality grading in this patient population. This may explain the limitation in interobserver agreement findings related to image quality. While our results only found fair interobserver agreement regarding image quality, full agreement between the two observers was noted in most of the images. Other limitations of this study included those inherent to a retrospective, non-controlled, non-randomized study design, the small sample size, and no defined imaging acquisition guidelines or protocols. The consecutive enrollment strategy employed in this study may have been a source of selection. In our study, Boston type I and type II Kpro patients were enrolled; therefore, these results may not extend to patients with other types of Kpro, such as in those with an osteo-odonto-keratoprosthesis. Owing to the retrospective study design and the lack of defined imaging protocols for this specific patient population, the results may not reflect the highest possible quality images. Thus, the true clinical utility of UWF imaging in Kpro patients may be understated based on the results of this study. Limitations associated with the Optos scanning laser ophthalmoscope imaging system include the need for a trained technician, imaging artifacts associated with the keratoprosthesis, and patient-specific fixation difficulties resulting in the need to acquire multiple images during a single imaging session.

Despite these limitations, the results of this study are in concordance with past studies. While the sample size was relatively small, the number of enrolled eyes was greater than those enrolled in previous studies. This study also adds evidence that UWF imaging can be used as a rapid and reliable method to serially image the posterior segment and may provide clinically meaningful findings in both type I and type II Kpro patients. UWF imaging serves as a valuable tool for the physician to monitor and manage these challenging patients.

## Conclusions

In summary, UWF imaging provided adequate visualization of the posterior segment in Kpro implanted patients. Importantly, this imaging modality allowed noninvasive longitudinal monitoring of retinal and optic nerve clinical pathology in this patient population. UWF imaging demonstrated clinical utility in this group of patients with prevalent posterior segment pathology.

## Data Availability

The data used in this study are available from the corresponding author upon reasonable request.

## Abbreviations

Kpro: Keratoprosthesis
UWF: Ultra-wide field
κ: Kappa statistic coefficient
BCL: Bandage contact lens
RPM: Retroprosthetic membrane
SD: Standard deviation
CI: Confidence interval
CME: Cystoid macular edema
AMD: Age-related macular degeneration
PPV: Pars plana vitrectomy
